# Quantifying Spatial Variability of Selected Soil Trace Elements and Their Scaling Relationships Using Multifractal Techniques

**DOI:** 10.1371/journal.pone.0069326

**Published:** 2013-07-09

**Authors:** Fasheng Zhang, Guanghua Yin, Zhenying Wang, Neil McLaughlin, Xiaoyuan Geng, Zuoxin Liu

**Affiliations:** 1 Institute of Applied Ecology, Chinese Academy of Sciences, Shenyang, China; 2 Eastern Cereal and Oilseed Research Centre, Agriculture and Agri-Food Canada, Ottawa, Canada; Medical University of Graz, Austria

## Abstract

Multifractal techniques were utilized to quantify the spatial variability of selected soil trace elements and their scaling relationships in a 10.24-ha agricultural field in northeast China. 1024 soil samples were collected from the field and available Fe, Mn, Cu and Zn were measured in each sample. Descriptive results showed that Mn deficiencies were widespread throughout the field while Fe and Zn deficiencies tended to occur in patches. By estimating single multifractal spectra, we found that available Fe, Cu and Zn in the study soils exhibited high spatial variability and the existence of anomalies ([*α*(*q*)_max_−*α*(*q*)_min_]≥0.54), whereas available Mn had a relatively uniform distribution ([*α*(*q*)_max_−*α*(*q*)_min_]≈0.10). The joint multifractal spectra revealed that the strong positive relationships (*r*≥0.86, *P*<0.001) among available Fe, Cu and Zn were all valid across a wider range of scales and over the full range of data values, whereas available Mn was weakly related to available Fe and Zn (*r*≥0.18, *P*<0.01) but not related to available Cu (*r* = −0.03, *P* = 0.40). These results show that the variability and singularities of selected soil trace elements as well as their scaling relationships can be characterized by single and joint multifractal parameters. The findings presented in this study could be extended to predict selected soil trace elements at larger regional scales with the aid of geographic information systems.

## Introduction

Although trace elements are needed by plants in only very small quantities [1], [2], their deficiencies in soils may result in severe plant malnutrition as well as animal and human health problems [3], [4]. Quantifying the spatial variability of soil trace elements enables prediction of trace elements at intermediate locations where measurements have not been made [5]. The thematic map produced by the prediction is critical to understanding, assessing and managing trace element-deficient soils [6].

Plant availability of trace elements in soils have been shown to have a high degree of spatial variability [7], [8]. This arises mainly from the existence of anomalies in their distributions, which is a consequence of local extreme soil and weather conditions [3] such as alkalinity, soil infertility, erosion, drought, etc. From a practical standpoint, anomalies may carry valuable information for identifying specific trace element deficiencies. Including anomalies is essential when developing models that characterize the spatial variability of soil trace elements.

Under an assumption of intrinsic stationarity, the semivariogram has been the most common method for quantifying the spatial variability of soil trace elements [7]–[11]. In modern geostatistics, anomalies in a trace element dataset are usually transformed or eliminated in advance to avoid erratic behavior of a semivariogram [12]. As a second-order moment statistic, semivariogram cannot differentiate subtle variations in a trace element dataset. These facts reveal that current semivariogram models may not be able to fully characterize the spatial variability of soil trace elements, especially the singularities of anomalies.

Over a given area, plant availability of soil trace elements is commonly associated with similar soil and environmental conditions. An understanding of the relationships among soil trace elements can help in the analysis of the underlying processes controlling their spatial variability [7], [8]. Thus, there is a need to examine the spatial variability of soil trace elements in a way that takes account of the relationships among them.

Factors and processes affecting soil properties including plant availability of trace elements operate at different spatial scales and influence these properties in different ways [9]. Relationships among soil trace elements may be heterogeneous across nested spatial scales. Although correlation analysis can quantify the relationship among soil trace elements at the measured scale, this relationship may not be valid at other spatial scales. As such, relationships among soil trace elements across a range of spatial scales, i.e., scaling relationships, are needed.

Over the past three decades, single multifractal spectrum has been applied in soil science to quantify the spatial variability of soil properties [13]–[18] including pH, organic matter content, cation exchange capacity, macro- and moderate-nutrient content, texture, structure, hydraulic properties, etc. The relationships among these properties and their associations with other environmental variables across spatial scales have also been investigated by researchers using a joint multifractal spectrum [18]–[20]. To date, however, studies examining spatial variability and singularity of soil trace elements and their scaling relationships using multifractal techniques are still rare [21]. Usually, the spatial distributions of pH, organic matter content, macro-nutrient content, texture, and other basic soil properties are relatively uniform. Geostatistical methods are able to characterize their spatial variability well and predict them with high accuracy. However, the distribution of soil trace elements is quite different from that of other soil properties, and is often characterized by high spatial variability and anomalies that are accompanied by singularities. As previously mentioned, semivariogram analysis and correlation analysis may not be able to completely characterize the singularity of soil trace elements and their scaling relationships. Panahi and Cheng pointed out that an exploratory dataset with anomalies may be more likely to follow a multifractal rather than a normal or lognormal distribution [22]. Meanwhile, the multifractal techniques have long been known to be able to characterize singularities and scaling relationships. Thus, the application of multifractal techniques in the analysis of soil spatial variability and their scaling relationships may be more appropriate for spatial analysis of soil trace elements rather than for other soil properties.

The objective of this study was therefore to examine whether the spatial distribution of selected soil trace elements in an agricultural field in northeast China exhibits a multifractal property, and if so, to quantify their spatial variability and scaling relationships using multifractal techniques. We also discuss the practical significance of the findings for prediction of selected soil trace elements.

## Theory of Multifractal Analysis

### Single Multifractal Spectrum

For a spatial dataset, there are several methods for estimating a single multifractal spectrum (e.g., the moment method, Rényi method). Here we used the moment method discussed by Agterberg [23], which is much easier to apply yet still provides accurate results.

Using this method, a normalized variable, *P_i_*(*ε*), is defined first according to:
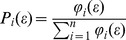
where *φ_i_*(*ε*) is the measured value of variable *φ* in the *i* ^th^ segment of scale *ε* in a plot, and *n* is the number of segments of scale *ε* over the whole plot. A partition function, *χ_q_*(*ε*), which varies with the moment order *q* and scale *ε*, is then calculated as:



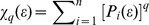



For a spatial dataset, the power-law relationship between the partition function and scale is used to determine whether or not it exhibits a multifractal property. This power-law relationship can be confirmed if the logarithm of the partition function varies linearly against a logarithm of scale with a slope of the mass exponent, *τ*(*q*):
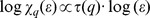



By plotting log*χ_q_*(*ε*) against log*ε* at many moment orders *q*, we compute the generalized fractal dimension, *D*(*q*):
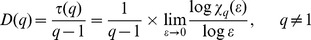


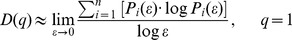



Then the Hölder exponent (i.e., singularity index), *α*(*q*), with respect to variable *φ*, is computed by Legendre transformation: 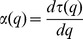



The fractal dimension, ƒ(*q*), with respect to variable *φ*, is given as:
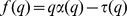



Plotting ƒ(*q*) against *α*(*q*) produces the so-called single multifractal spectrum. *α*(*q*) can be interpreted as an index of crowding of variable *φ* at the moment order *q* when *ε* approaches zero. ƒ(*q*) is the fractal dimension of the subsets of variable *φ* having specific *α*(*q*). Thus, the abundance of *α*(*q*) reflects the extent of spatial variability in variable *φ*. More *α*(*q*) (i.e., wider spectrum) represent greater spatial variability in distribution of variable *φ* and vice versa. In addition, with the amplification of high positive or negative *q* values, the spatial variability controlled by the higher or lower values of variable *φ* is separated from each other and represented by the *α*(*q*) in the left and right parts of the single multifractal spectrum, respectively. In particular, the *α*(*q*)_max_ and *α*(*q*)_min_ correspond to the singularity strength of the lowest and highest values of variable *φ*, respectively. The single multifractal spectrum therefore can detect a spatial pattern and separate anomalous variation from the background variation [13], [23].

Chhabra and Jensen [24] proposed a method to avoid the difficult task of estimating *α*(*q*) by Legendre transformation. In the Chhabra and Jensen method, we define another normalized variable, *δ_i_*(*q*,*ε*): 
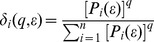



Then the singularity index and fractal dimension are computed respectively as:
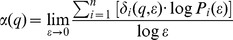


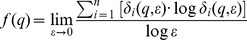



### Joint Multifractal Spectrum

For coexisting variables, we need to extend the single multifractal spectrum of each variable to a joint multifractal spectrum for two variables [25]. Two normalized variables, *P_i_*(*ε*) and *Q_i_*(*ε*), are first defined respectively for variables *φ* and *μ*. Then the joint normalized variable for variables *φ* and *μ*, *δ_i_*(*q*,*t*,*ε*), is calculated as: 
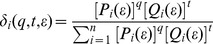
where *q* and *t* are both moment orders and *ε* is the scale. The singularity indices, *α*(*q*,*t*) and *β*(*q*,*t*), with respect to variables *φ* and *μ*, are computed respectively as:



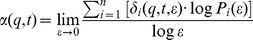


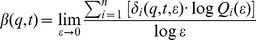



Then the joint fractal dimension, ƒ(*q*,*t*), with respect to variables *φ* and *μ*, is given as:
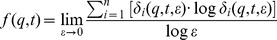



Plotting ƒ(*q*,*t*) against *α*(*q*,*t*) and *β*(*q*,*t*) produces the so-called joint multifractal spectrum, contours of which describe the scaling relationships between variables *φ* and *μ*. For positive scaling relationships, the joint distribution of higher *α*(*q*,*t*) and *β*(*q*,*t*) values at the upper right part of the joint multifractal spectrum indicates the positive correlation between lower values of variables *φ* and *μ*, while the joint distribution of lower *α*(*q*,*t*) and *β*(*q*,*t*) values at the lower left part indicates the positive correlation between higher values of variables *φ* and *μ*. For negative scaling relationships, higher *α*(*q*,*t*) values correspond to lower *β*(*q*,*t*) values and vice versa at the diagonal parts of the joint multifractal spectrum, which indicates that lower and higher values of variables *φ* and *μ* are distributed together. If variables *φ* and *μ* are not correlated, there will be no correlation between *α*(*q*,*t*) and *β*(*q*,*t*) [25], [26].

## Materials and Methods

### Site and Soil

The study site is located in Fuxin County in northeast China (42°08′14′′ N, 121°44′21′′ E). The climate is typical of a warm temperate zone with continental monsoons, hot summers and cold winters. The mean annual temperature is 7.2 degrees Celsius with an average of 2868 hrs of annual sunshine. This region is a typical dryland farming area with an average annual precipitation of 480 mm, over 60% of which occurs between June and August (SWCP I soil moisture sensor & WITU-WS weather station, WITU Technologies, Inc., China).

The main agricultural soil in the region is cinnamon soil which develops through a combination of calcium carbonate leaching, illuviation and humification. It is characterized by a thin humus layer and medium or thick solum. Soil pH (H_2_O) is 7.5−8.5 and soil organic matter content is 10.2 g·kg^−1^. The cation exchange capacity is 19.2 meq/100 g, and mean concentrations of available Fe, Mn, Cu, and Zn are 9.9 mg·kg^−1^, 36 mg·kg^−1^, 1.32 mg·kg^−1^, and 1 mg·kg^−1^, respectively (The Second National Soil Survey of China, 1985).

### Data Collection and Analysis

A total of 1024 soil samples were collected from a flat agricultural field of 10.24-ha (320 m by 320 m) in Matiyingzi Village, 10 km from the center of Fuxin County, in April 2008 (before annual planting). The soils were sampled over the 20-cm depth within a 10-m grid. The samples were taken with a stainless steel sampler and brought to the laboratory. After removing visible plant debris and air drying, soil samples were ground to pass through a 2-mm plastic sieve. Available Fe, Mn, Cu and Zn in each sample were measured by a DTPA (diethylene triamine pentaacetic acid) soil test [27]. The extractant consisted of 0.005 M DTPA, 0.1 M TEA (triethanolamine), and 0.01 M CaCl_2_, with a pH of 7.3. Hooda and Alloway reported that DTPA method could provide accurate information about plant availability of trace elements in soils [28]. All trace element concentrations were determined by inductively coupled plasma mass spectrometry (Perkin Elmer Optima 3000). All necessary permits were obtained for the described field studies. The land user and owner, Mingfu Zhang, approved the field-work activities at each sampling location. The field examined in this study is not protected in any way, and the study did not involve any endangered or protected species.

A series of five scales were superimposed on the sample plot: 10-m pixel, 20-m pixel, 40-m pixel, 80-m pixel, and 160-m pixel with 1,024, 256, 64, 16 and 4 samples, respectively. The dataset for the minimum scale was the measured trace element data in the initial 10-m pixel grid, while the dataset for the other scales was the average of the measured trace element data of all initial 10-m pixel grids included within the larger pixel in the respective grid.

The single and joint multifractal spectra parameters for selected soil trace element data were calculated using the equations in the part of theory of multifractal analysis by an Office VBA project and were presented in graphs by SigmaPlot 10.0. The correlation analysis between *α*(*q*,*t*) and *β*(*q*,*t*) was conducted using SPSS 16.0.

## Results

### Descriptive Statistics

A summary of descriptive statistics for selected trace elements in the study soils is presented in Table 1. Available Fe, Cu and Zn varied to a high degree with coefficient of variation (CV) of 82.7%, 41.7% and 89.5%, respectively. The CV is very sensitive to the anomalies in a dataset and high CV's for available Fe, Cu and Zn data suggest that there must be some significant anomalous values in their distributions. Available Mn had a relatively uniform distribution with a CV of only 9.5%. Based on the thresholds recommended by Liu [29], we found that Mn deficiency was widely present throughout the field. Although Fe and Zn deficiencies were not widespread, 25.5% of soil samples were deficient in available Fe, and 13.3% were deficient in available Zn. This indicated that Fe and Zn deficiencies tended to occur in patches rather than throughout the field. Only a very limited percentage of soil samples were deficient in available Cu.

**Table 1:** Descriptive statistics for selected soil trace elements in the study agricultural field of northeast China.

Trace elements	Min† (mg•kg^−1^)	Max (mg•kg^−1^)	Mean (mg•kg^−1^)	CV	Deficiency‡
Fe	0.30	73.73	18.93	82.7%	25.5%
Mn	25.53	91.11	69.27	9.5%	100%
Cu	0.18	5.27	1.34	41.7%	1.4%
Zn	0.02	14.81	2.48	89.5%	13.3%

† Min, Max and CV are abbreviations for minimum, maximum and coefficient of variation.

‡ Critical levels for available Fe, Mn, Cu and Zn recommended by Liu [29] are 4.5 mg•kg^−1^, 100 mg•kg^−1^, 0.3 mg•kg^−1^ and 1.0 mg•kg^−1^ (DTPA extracted). The concentrations of available Fe, Mn, Cu and Zn under the critical levels are recognized as deficiencies.

### Empirical Tests of Multifractality

**Figure 1:**
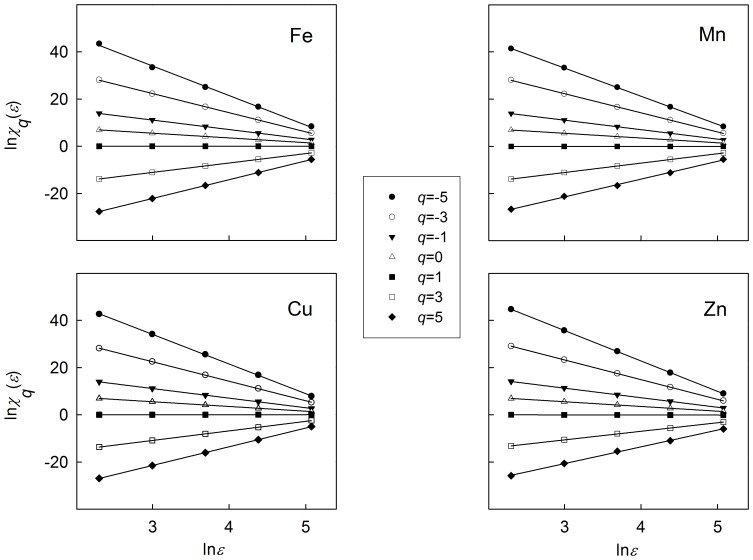
Logarithms of partition functions of selected soil trace elements plotted against scales. The *R* ^2^ values of the linear fit for all *q* considered were always greater than 0.99.

Scatter plots and their fitted relationships between the logarithm of partition function and the logarithm of superimposed scale for selected soil trace elements are shown in Figure 1. The coefficients of determination *R* ^2^ for all linear regression lines were larger than 0.99 (*F*-test, *P*<0.001) which confirms the assumption of the existence of the power-law relationship between partition function and scale. This finding revealed that the spatial distribution of available Fe, Mn, Cu and Zn in the study soils exhibited a multifractal property. Hence, multifractal techniques can be used to characterize the spatial variability of selected soil trace elements and their scaling relationships.

**Figure 2:**
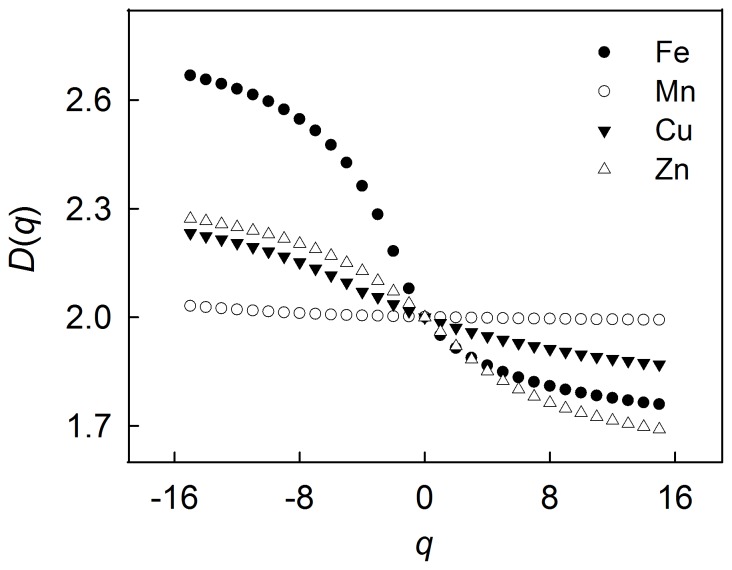
General fractal dimension *D*(*q*) for selected soil trace elements at −15 to 15 range of *q* evaluated at 1 increment.

Variables showing a multifractal property usually have a *D*(*q*) that demonstrates a monotonic and nonlinear trend with *q* values. This pattern can be observed when examining the trend of *D*(*q*) for available Fe, Cu and Zn in Figure 2. The *D*(*q*) values for available Fe, Cu and Zn decreased markedly as *q* values increased. This is a typical characteristic of a multifractal system, and also indicated that the multifractal property observed here was strong. In contrast, the *D*(*q*) values for available Mn decreased very slowly against *q* values and showed small deviations from the capacity dimension of the sample plot (i.e., 2). This revealed that the distribution of available Mn can be represented by a monofractal or a weak multifractal. Note that a monofractal is a special case of a multifractal.

### Variability and Singularity of Soil Trace Elements

**Figure 3:**
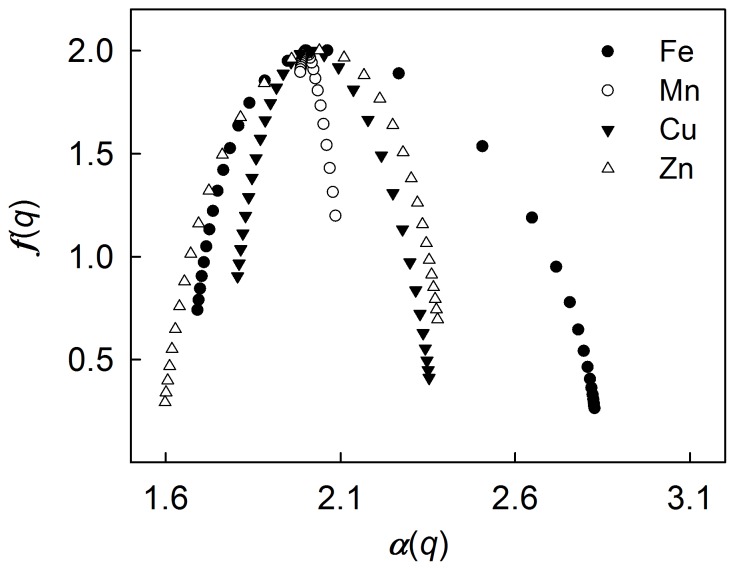
Single multifractal spectrum for selected soil trace elements at −15 to 15 range of *q* evaluated at 1 increment.

To examine the spatial variability of selected soil trace elements, their single multifractal spectra were constructed and shown in Figure 3. The single multifractal spectra for available Fe, Cu and Zn all had large widths ([*α*(*q*)_max_−*α*(*q*)_min_]≥0.54). This indicated that available Fe, Cu and Zn had quite high spatial variability in studied soils. To characterize such high spatial variability of available Fe, Cu and Zn, a large number of singularity indices ranging from 1.69 to 2.83, 1.81 to 2.35, and 1.60 to 2.38 respectively were needed. In contrast, the single multifractal spectrum of available Mn had a very narrow width ([*α*(*q*)_max_−*α*(*q*)_min_]≈0.10). This indicated that the spatial variability of available Mn was relatively low. Such low spatial variability can be characterized by a very small set of singularity indices, ranging from 1.99 to 2.09.

The single multifractal spectra of available Fe, Mn, Cu and Zn all had asymmetric tails on either the right or left part (Figure 3), which is another typical characteristic of a multifractal property. The left and right parts of a single multifractal spectrum respectively represent the higher and lower values of a dataset, emphasized by high positive and negative *q* values. The long left and right tails of the single multifractal spectra for available Fe, Cu and Zn were therefore the result of both extremely high and low values in their distributions. The singularities of these anomalies were characterized by the corresponding singularity indices in the single multifractal spectra of available Fe, Cu and Zn. The single multifractal spectrum of available Mn had a relatively long tail only on the right part. This indicated that there were no anomalies but just some relatively low values in the distribution of available Mn in the study soils.

### Scaling Relationships among Soil Trace Elements

**Figure 4:**
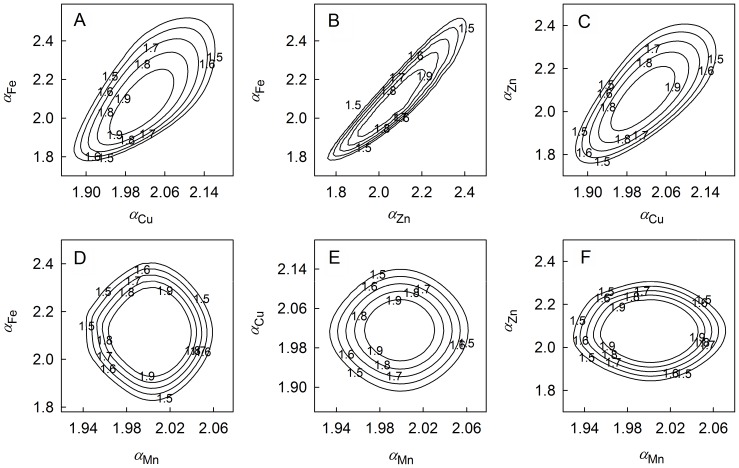
Joint multifractal spectrum for selected soil trace elements at −15 to 15 range of *q* evaluated at 1 increment.

The relationships among selected soil trace elements across the spatial scales superimposed on the sample plot were analyzed using the joint multifractal spectra (Figure 4). In general, there were strong positive scaling relationships among available Fe, Cu and Zn, which were evident from the compact joint distribution of their singularity indices at the upper right and lower left parts of their joint multifractal spectra (Figures 4A, 4B and 4C). The contours in Figure 4B were tightly concentrated in one bundle. This indicated that there were strong positive relationships between available Fe and Zn for both higher and lower values across the superimposed spatial scales. With a small diagonal stretch at the upper right, Figure 4A and Figure 4C had a similar pattern of joint distribution of singularity indices as Figure 4B. This indicated that strong positive relationships also existed between available Cu and available Fe and Zn over a wider spatial scale and for the entire range of data values. In Figures 4D, 4E and 4F, the wide stretch of diagonal contours showed that the singularity indices of available Mn was not correlated tightly with that of available Fe, Cu and Zn. This suggested that available Mn may not be associated, or may be weakly associated, with available Fe, Cu and Zn over the range of measured concentrations and superimposed spatial scales.

To further confirm the scaling relationships among selected soil trace elements, the Pearson correlation coefficients (*r*) between their singularity indices were analyzed (Table 2). The singularity indices between available Fe and Zn had the highest positive correlation coefficient (*r* = 0.98, *P*<0.001). The positive correlation coefficients between the singularity indices of available Fe versus available Cu and available Zn versus available Cu were relatively high as well (*r* = 0.87 and 0.86, *P*<0.001). These results indicated that the positive correlation relationships among available Fe, Cu and Zn were all very strong across superimposed spatial scales. Although the associations between singularity indices of available Fe versus available Mn and available Zn versus available Mn were also significant, their correlation coefficients (*r* = 0.19 and 0.18, *P*<0.01) were relatively low compared to those among available Fe, Cu and Zn. This suggested the positive correlations between available Mn and available Fe and Zn were weak. The correlation coefficient between singularity indices of available Cu and Mn was not significant (*r* = −0.03, *P* = 0.40), which implied that available Cu and Mn were not correlated across superimposed spatial scales. The strong scaling relationships among available Fe, Cu and Zn were likely due to a common controlling process, which was quite different from that for available Mn.

**Table 2:** Pearson correlation coefficients (*r*) between singularity indices of joint multifractal spectra for selected soil trace elements.

	Fe	Mn	Cu
Mn	0.19 (*P*<0.01)		
Cu	0.87 (*P*<0.001)	−0.03 (*P* = 0.40)	
Zn	0.98 (*P*<0.001)	0.18 (*P*<0.01)	0.86 (*P*<0.001)

## Discussion

The influences of factors in the CLORPT function or the SCORPAN model on the variations of selected soil trace elements should be relatively consistent over a flat 10.24-ha level field [30], [31]. In most soils, available Fe, Mn, Cu and Zn are not directly related to their total contents, but are more related to other soil characteristics such as pH, redox potential, organic matter content, cation exchange capacity, and texture [32]. In agricultural soils, long-term intensive agricultural practices also add another layer of complexity on the distribution of available Fe, Mn, Cu and Zn. Kravchenko et al. [13], Perrier and Bird [14], Caniego et al. [15], Guan et al. [16] and Perrier et al. [33] have reported that pH, redox potential, organic matter content, cation exchange capacity, texture and structure interact with each other and are prone to lead to a nonlinear soil system in which multifractal properties are expressed. The multifractal nature of available Fe, Mn, Cu and Zn observed in this study can be attributed to the complexity of this system.

The spatial variability of selected soil trace elements characterized by single multifractal spectrum was partly consistent with the result of CV analysis. However, the results based on the single multifractal spectrum were obtained over a wider range of spatial scales and moment orders and better reflected the true spatial variability of selected soil trace elements. The single multifractal spectrum also captured the detailed singularity strength of anomalies in available Fe, Mn, Cu and Zn data while the joint multifractal spectrum characterized the scaling relationships among them. These two points demonstrate that multifractal techniques present unique advantages over CV and semivariogram analyses.

Knowledge of soil spatial variability is a prerequisite needed to predict soil properties at unsampled locations. In this study, the spatial variability and singularity of selected soil trace elements quantified by the parameters of single multifractal spectra would have practical significance for prediction of these trace elements. Appropriate prediction procedures for variables with high spatial variability and a large amount of singularity indices are different than that for those with low spatial variability and limited singularity indices. This implication has been summarized in the study by Cheng [34] and justified by Cheng [35] and Xie et al. [36]. In our study, available Mn had a narrow multifractal spectrum with a limited number of singularity indices. Its spatial variability was very low and there were no anomalies within its spatial distribution. For a variable with those distribution characteritics and spatial autocorrelation, a simple prediction procedure such as an ordinary Kriging interpolation may be appropriate. In contrast, available Fe, Cu and Zn in this study's soils had wide multifractal spectra with many singularity indices both at *α*(*q*)>2 and *α*(*q*)<2. Their spatial variability was high with both extremely high and extremely low values in their spatial distributions. Under these circumstances, it is possible, but difficult, to apply a prediction procedure. For example, *α*(*q*)>2 implied that there were some specific locations with extremely low concentrations of available Fe, Cu and Zn. An ordinary interpolation will smooth out the extremely low values of available Fe, Cu and Zn, resulting in the loss of valuable information for identifying Fe, Cu and Zn deficiencies. In this case, incorporating singularity indices *α*(*q*)>2 into the prediction procedure can help preserve the extremely low values of available Fe, Cu and Zn. Similarly, *α*(*q*)<2 is also needed when predicting available Fe, Cu and Zn with extremely high values at specific locations.

Recent studies have delineated the spatial distribution of selected trace elements in soils using their relationships with environmental data, but those relationships were observed at only a single scale and may not be valid at other scales. Across a regional soil landscape, factors and processes impacting the plant availability of soil trace elements are expected to be much more complicated and heterogeneous than that of the 10.24-ha agricultural field observed in this study. The major controlling processes for different soil trace elements may differ at specific spatial scales, which would further impact the spatial variability of soil trace elements. The results of joint multifractal analysis in this study had demonstrated the ability of joint multifractal spectrum in characterizing the scaling relationships among selected soil trace elements at the field scale. It is well-known that fractals and multifractals are ubiquitous in complex ecosystems [37]. Further study is needed to evaluate the technique in modeling the scaling relationships of soil trace elements at larger regional scales where their variability in relation to the local environments would be expected. Using scale-specific relationships future researchers should be able to improve models when predicting soil trace elements with auxiliary environmental data.

In recent years, geographic information systems (GIS) have facilitated the process of spatial analysis and computation. If further research demonstrates that the multifractal phenomenon exists over larger regional scales, the findings in this study could be incorporated into GIS to improve the prediction of selected trace elements over a range of scales for optimizing soil use and management.

## Conclusions

Spatial variability of available Fe, Mn, Cu and Zn and their scaling relationships were examined using multifractal techniques for a 10.24-ha agricultural field in northeast China. The distribution of selected soil trace elements exhibited a multifractal property. The variability and singularities of available Fe, Cu and Zn in the study soils needed to be quantified by multifractal spectra, whereas that of available Mn can be characterized by a monofractal or a weak multifractal spectrum. The joint multifractal spectra and the Pearson correlation analysis of their singularity indices captured the relationships among selected soil trace elements across a wider range of spatial scale and over the full range of data values.

The use of single and joint multifractal parameters has potential to enhance the prediction of selected soil trace elements in future research. We are currently examining the multifractality of soil trace elements at the county scale and are building a model that incorporates the scaling relationships between selected soil trace elements and environments into a prediction procedure. Such studies are essential to validate the applicability and extension of the findings presented here.
